# Isospecific adenine DNA methyltransferases show distinct preferences towards DNA substrates

**DOI:** 10.1038/s41598-018-26434-0

**Published:** 2018-05-29

**Authors:** Ewa Wons, Iwona Mruk, Tadeusz Kaczorowski

**Affiliations:** 1 Department of Microbiology, Faculty of Biology, University of Gdańsk, Wita Stwosza 59, Gdansk, 80-308 Poland; 2 Laboratory of Extremophiles Biology, Department of Microbiology, Faculty of Biology, University of Gdańsk, Wita Stwosza 59, Gdansk, 80-308 Poland

## Abstract

Here, we report results on systematic analysis of DNA substrate preferences of three N6-adenine β-class DNA methyltransferases that are part of the type II restriction-modification systems. The studied enzymes were: M.EcoVIII, M.HindIII and M.LlaCI, which although found in phylogenetically distant bacteria (γ-proteobacteria and low-GC Gram-positive bacteria), recognize the same palindromic specific sequence 5′-AAGCTT-3′ and catalyze formation of N6-methyladenine at the first A-residue. As expected overall the enzymes share the most analyzed features, but they show also some distinct differences in substrate recognition. Therefore DNA methylation reactions were carried out not only under standard, but also under relaxed conditions using DMSO or glycerol. We found that all of these enzymes preferred DNA containing a hemimethylated target site, but differ in modification of ssDNA, especially more pronounced for M.EcoVIII under relaxed conditions. In these conditions they also have shown varied preferences toward secondary sites, which differ by one nucleotide from specific sequence. They preferred sequences with substitutions at the 1^st^ (A^1^ → G/C) and at the 2^nd^ position (A^2^ → C), while sites with substitutions at the 3^rd^ position (G^3^ → A/C) were modified less efficiently. Kinetic parameters of the methylation reaction carried out by M.EcoVIII were determined. Methylation efficiency (k_cat_/K_m_) of secondary sites was 4.5–10 times lower when compared to the unmethylated specific sequences, whilst efficiency observed for the hemimethylated substrate was almost 4.5 times greater. We also observed a distinct effect of analyzed enzymes on unspecific interaction with DNA phosphate backbone. We concluded that for all three enzymes the most critical is the phosphodiester bond between G^3^-C^4^ nucleotides at the center of the target site.

## Introduction

Recognition by a protein of a specific nucleotide sequence in DNA is a key element in many biological processes, including regulation of gene expression, replication and recombination of DNA. The mechanisms of such interactions with target sequences have been extensively studied, yet they are still incompletely understood. Enzymes constituting type II restriction-modification systems are known for their unusual accuracy in recognizing relatively short (4–8 nt) nucleotide sequences^1^. Several possible roles of RM systems have been postulated, but the major ones seem to operate for bacterial protection against invasive DNAs^2,3^. Both of the RM system type II components, the restriction endonuclease (R/ENase) and the DNA methyltransferase (M/MTase), are independent in their action. Despite different function and very low similarity at their amino acid sequences, they act in a sequence-specific manner to recognize the same target nucleotide sequence^1,4^. Restriction endonuclease recognizes a specific sequence and digests it, while DNA MTase modifies adenine or cytosine within the target site and therefore protects bacterial DNA from cleavage by a cognate restriction enzyme. Although a typical DNA MTase exerts high specificity toward its target site, it also exhibits a certain level of promiscuity or relaxed activity. Such action results in DNA methylation of the secondary sites (off-target) which are by one or more bases different (star sites) from the canonical target sequence^5–11^. Other MTases, which do not use DNA as a substrate, seem to share such promiscuity as well^12–14^.

So far over 300 target sequences have been determined^15^. In addition, a great number of enzymes of different origin but which share the function and the recognition sequences has been identified. These isospecific enzymes often come from phylogenetically distant bacteria (Gram-negative and Gram-positive). The purpose of our studies was to test if these enzymes of the same DNA substrate specificity somehow differ in mechanisms of DNA recognition. Such analysis may provide a deeper understanding of molecular processes responsible for the specific DNA-protein interactions and may pave the way for studies on evolutionary aspects of target recognition.

**Figure 1:**
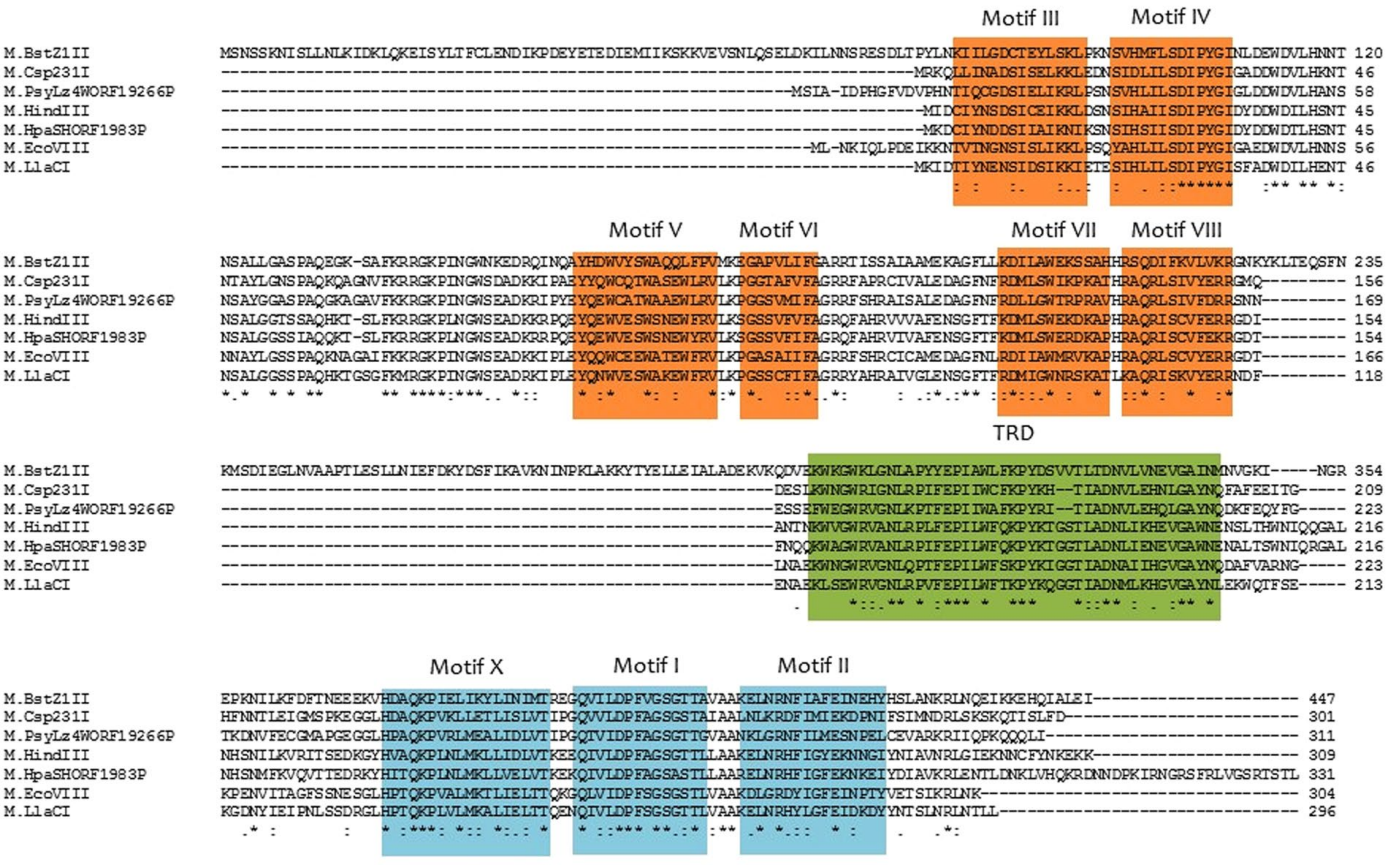
Comparison of amino acid sequences of DNA methyltransferases recognizing the 5′-AAGCTT-3′ specific sequence. The conserved motifs characteristic for N6-adenine β-class MTases are marked. Motifs X, I and II bind AdoMet, III-VIII are responsible for catalysis, TRD recognizes the specific sequence. The accession numbers of the nucleotide sequences of the M.BstZ1II, M.Csp231I, M.PsyLz4WORF19266P, M.HindIII, M.HpaSHORF1983P, M.EcoVIII and M.LlaCI genes that have been deposited in the GenBank database are AY789018, AY787793, CP017432, L15391, CP001321, AF158026, and AJ002064, respectively.

In our work we focused on three bacterial isospecific DNA MTases that are part of type II RM systems (Fig. 1). The tested enzymes were: M.EcoVIII from *Escherichia coli* E1585-68^16–21^, M.HindIII from *Haemophilus influenzae* Rd^22,23^ and M.LlaCI from *Lactococcus lactis* subs. *cremonis* W15^24^. Their amino acid sequences reveal presence of nine sequence motifs conserved among the N6-adenine β-class MTases^25^. They all recognize the 5′-AAGCTT-3′ target sequence and modify the first adenine residue to N6-methyladenine using S-adenosyl-L-methionine as the methyl group donor (AdoMet). Although exhibiting the same substrate specificity, those three enzymes have different properties, and therefore are an excellent model to study molecular basis of target recognition. Comparative analysis of their amino acid sequences revealed that their identity ranges from 50 to 62%, and the most similar are M.EcoVIII and M.LlaCI (62%), while the least similar are M.EcoVIII and M.HindIII (50%). It has been shown, that only M.LlaCI is able to cross-react with anti-M.EcoVIII polyclonal antibodies, which reflects common epitopes between those two enzymes^16^. Furthermore, M.LlaCI exists in solution predominantly as a dimer, which is a rather exceptional feature, as the great majority of DNA methyltransferases, including M.EcoVIII and M.HindIII, function as monomers. Moreover, these enzymes were found to exhibit diverse sensitivity to Mg^2+^ ions^26^. Two tested proteins – M.LlaCI and M.EcoVIII, also seem to specifically modify the DNA strand in a DNA/RNA hybrid^27^.

In this work, we analyzed isospecific MTases in their response to standard and relaxed reaction conditions. As a result, we were able to show their distinct preferences towards DNA substrates.

## Materials and Methods

### Oligonucleotides

Standard oligonucleotides were synthesized at the Institute of Biochemistry and Biophysics, Polish Academy of Sciences (Poland). Oligonucleotide with a methylated target sequence was obtained from GenoTech (South Korea). For preparation of a specific substrate, equimolar aliquots of complementary oligonucleotides were suspended in TE buffer and heated to 95 °C, followed by slow cooling to 4 °C to ensure complete annealing.

### Enzymes and DNA methylation assay

DNA MTases were purified as described previously^16,27^. MTase activity measurements were based on monitoring transfer of the radioactively labelled methyl groups from [^3^H]AdoMet to DNA. Methylation was performed in 20 µl reaction mixtures containing 0.15 µM double-stranded (ds) oligonucleotide, 10 mM Tris-HCl pH 7.0, 0.2 µM [methyl-^3^H]SAM (69.5 Ci/mmol; Amersham) and the enzyme (0.26 µM). Unless otherwise indicated, the reaction was carried out for 1 h at 37 °C and then stopped by adding two volumes (40 µl) of absolute ethanol and 0.1 volume (2 µl) of 3 M sodium acetate, pH 4.8 (Sigma-Aldrich) and subsequently precipitated overnight. Then, the samples were centrifuged (10 000 *g*, 40 min, 4 °C) and the pellet was washed with 0.7 ml of 70% ethanol, harvested by centrifugation (10 000 *g*, 10 min, 4 °C) and dried. Scintillation counting was performed using Beckman LS 6000TA counter to measure incorporated radioactivity. In addition, each time appropriate controls – negative and positive, were performed, and CPM value of negative control was always subtracted from all measurements and normalized to positive control if needed.

Enzyme steady-state kinetics in the presence of DNA substrates was monitored in 20µl reaction mixtures containing ds oligonucleotides (0.01–20 µM; Table 1), 1.76 µM [^3^H]AdoMet, and 135 nM of M.EcoVIII in 10 mM Tris-HCl, pH 7.0. Kinetic parameters in the presence of AdoMet were monitored in 20µl reaction mixtures containing 10 µM unmethylated ds oligo with the target site (AAGCTT1/AAGCTT2; Table 1), [^3^H]AdoMet (0.11–5.28 µM), and 135 nM of M.EcoVIII in 10 mM Tris-HCl, pH 7.0. The reaction was carried out for 5 min at 37 °C, then stopped and processed as described above. To induce star activity, the reaction mixtures were supplemented with DMSO or glycerol, as described in our previous report^27^.

**Table 1:** Oligonucleotides (29-mers) used to study methylation of the target specific sequence and secondary sites.

Oligonucleotides	5′-sequence-3′
AAGCTT1	TGCAGTCGCGAAGCTTGGTCACCTTGAGG
AAGCTT2	TGCCTCAAGGTGACCAAGCTTCGCGACTG
MAGCTT	TGCAGTCGCG^m^AAGCTTGGTCACCTTGAGG
CAGCTT1	TGCAGTCGCGCAGCTTGGTCACCTTGAGG
CAGCTT2	CCTCAAGGTGACCAAGCTGCGCGACTGCA
GAGCTT1	TGCAGTCGCGGAGCTTGGTCACCTTGAGG
GAGCTT2	CCTCAAGGTGACCAAGCTCCGCGACTGCA
TAGCTT1	TGCAGTCGCGTAGCTTGGTCACCTTGAGG
TAGCTT2	CCTCAAGGTGACCAAGCTACGCGACTGCA
ACGCTT1	TGCAGTCGCGACGCTTGGTCACCTTGAGG
ACGCTT2	CCTCAAGGTGACCAAGCGTCGCGACTGCA
AGGCTT1	TGCAGTCGCGAGGCTTGGTCACCTTGAGG
AGGCTT2	CCTCAAGGTGACCAAGCCTCGCGACTGCA
ATGCTT1	TGCAGTCGCGATGCTTGGTCACCTTGAGG
ATGCTT2	CCTCAAGGTGACCAAGCATCGCGACTGCA
AAACTT1	TGCAGTCGCGAAACTTGGTCACCTTGAGG
AAACTT2	CCTCAAGGTGACCAAGTTTCGCGACTGCA
AACCTT1	TGCAGTCGCGAACCTTGGTCACCTTGAGG
AACCTT2	CCTCAAGGTGACCAAGGTTCGCGACTGCA
AATCTT1	TGCAGTCGCGAATGTTGGTCACCTTGAGG
AATCTT2	CCTCAAGGTGACCAAGCTGCGCGACTGCA
CAGCTG1	TGCAGTCGCGCAGCTGGGTCACCTTGAGG
CAGCTG2	CCTCAAGGTGACCCAGCTGCGCGACTGCA
Control L1	GAGAGAGAGTGCAGTCGCGTCTCTCTCTC
Control L2	GAGAGAGAGACGCGACTGCACTCTCTCTC
Control R1	GAGAGAGGGTCACCTTGAGGCATCTCTCT
Control R2	AGAGAGATGCCTCAAGGTGACCCTCTCTC

Target nucleotide sequence is underlined.

### Preparation of DNA substrates lacking a single phosphodiester bond

**Figure 2:**
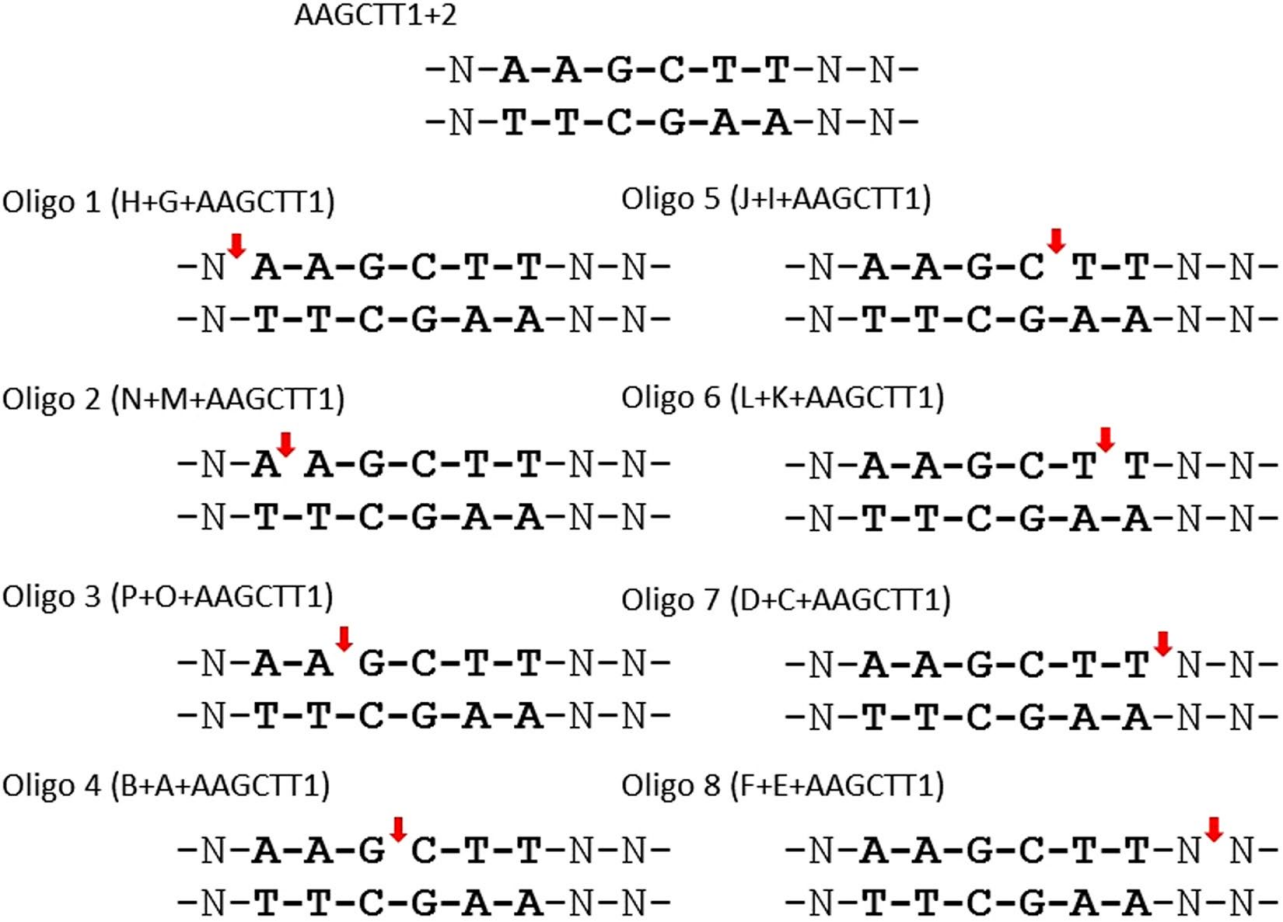
Schematic drawing of substrates used to estimate the importance of contacts created by tested DNA methyltransferases and the 5′-AAGCTT-3′ specific sequence (bold). Double-stranded oligos were prepared by annealing three DNA molecules, from which two (A-P) were complementary to AAGCTT1. Resulting substrates lacked a single phosphodiester bond (“gap” marked by arrow).

Annealing of oligo pairs from the A-P series (Table 2) with complementary oligonucleotide (AAGCTT1, Table 1) resulted in a set of ds substrates with different location of “a gap” within upper strand (Fig. 2). The completeness of substrate oligo assembly was tested in a DNA methylation assay. As control, ds oligo AAGCTT1/AAGCTT2 was used. Briefly, upper strand oligo (A, C, E, G, I, K, M, O) was phosphorylated with polynucleotide kinase (Thermo Scientific), and annealed along with its counterpart to the complementary template oligo (AAGCTT1). Then, T4 DNA ligase (EurX) and ATP were added to close the gap. Finally, substrate oligos were modified with isospecific MTases. We found that in each case the level of incorporation of radioactively labelled methyl groups was similar to that observed for control ds oligo (data not shown). For substrates with the target site localized in close proximity to the end of ds oligo, we used a set of complementary 29-mers: N0-1/N0-2, N1-1/N1-2, N2-1/N2-2 and N3-1/N3-2 (Table 2). These oligos differ by 1 nt in location of the target site. DNA methylation level was normalized to the one achieved with ds oligo AAGCTT1/AAGCTT2, with the target site localized 10 nt from the 5′ end and 13 nt from the 3′ end.

**Table 2:** Oligonucleotides used to test effect of target site position on efficiency of DNA methylation.

Oligonucleotides	5′-sequence-3′	Length
N0-1	AAGCTTCGCGTGCAGTGGTCACCTTGAGG	29
N0-2	CCTCAAGGTGACCACTGCACGCGAAGCTT	29
N1-1	GAAGCTTCGCGTGCAGTGGTCACCTTGAG	29
N1-2	CTCAAGGTGACCACTGCACGCGAAGCTTC	29
N2-1	GGAAGCTTCGCGTGCAGTGGTCACCTTGA	29
N2-2	TCAAGGTGACCACTGCACGCGAAGCTTCC	29
N3-1	GGGAAGCTTCGCGTGCAGTGGTCACCTTG	29
N3-2	CAAGGTGACCACTGCACGCGAAGCTTCCC	29
A	CTTCGCGACTGCA	13
B	CCTCAAGGTGACCAAG	16
C	CGCGACTGCA	10
D	CCTCAAGGTGACCAAGCTT	19
E	GCGCCTGCA	9
F	CCTCAAAGGTGACCAAGCTTC	10
G	AAGCTTCGCGACTGCA	16
H	CCTCAAGGTGACC	13
I	TTCGCGACTGCA	12
J	CCTCAAGGTGACCAAGC	17
K	TCGCGACTGCA	11
L	CCTCAAGGTGACCAAGCT	18
M	AGCTTCGCGACTGCA	15
N	CCTCAAGGTGACCA	14
O	GCTTCGCGACTGCA	14
P	CCTCAAGGTGACCAA	15

The ds oligos tested contained target site localized at various positions with respect to its 5′ end (N0-N3) or were deprived of a single phosphodiester bond within the target site or its proximity (A-P). The tested target site, or its part, is underlined.

### Data analysis

All enzyme activity data were averaged for at least three measurements. Kinetic data were analyzed using Sigma Plot 12.

## Results

### All three MTases prefer hemimethylated substrate, but differ in ssDNA modification

**Figure 3:**
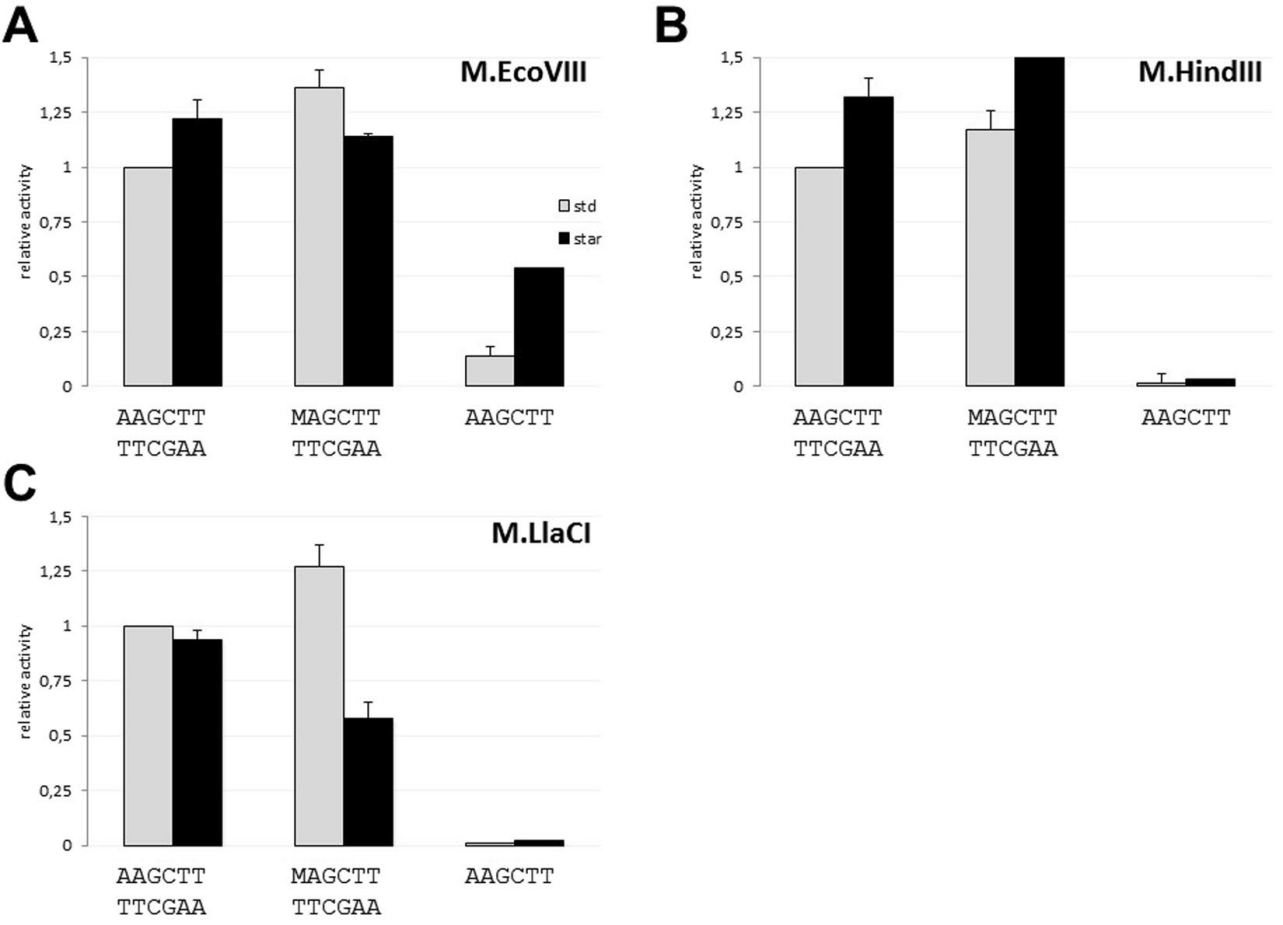
Analysis of DNA methyltransferase activity towards the 5′-AAGCTT-3′ specific sequence carried on ds-oligo with various methylation status (unmethylated or hemimethylated), or ss-oligo. M stands for N^6^A. Methylation was carried out by M.EcoVIII (**A**), M.HindIII (**B**), M.LlaCI (**C**) in standard (grey columns) or star activity (black columns) reaction mixtures. Methylation level was normalized to the one obtained in a standard reaction mixture with ds-unmethylated sequence (methylation level = 1). Note, methylation substrates differ in number of target sequence – one in case of hemimethylated (MAGCTT/TTCGAA) and ssAAGCTT, two in unmethylated oligo (AAGCTT/TTCGAA).

In order to determine substrate preferences of the analyzed MTases, ds oligonucleotides (29-mers, Table 1) that carried either unmethylated (oligo AAGCTT1/AAGCTT2, Table 1) or hemimethylated (oligo MAGCTT/AAGCTT2, Table 1) target site were used. We found that under standard conditions, all three MTases have shown a preference towards hemimethylated ds oligo which was modified 1.1–1.3 more efficiently when compared to the unmethylated control (Fig. 3). We have also noted that under star activity conditions (30% DMSO), modification activity towards the unmethylated substrate was 1.2–1.3-fold higher when compared to the one observed under standard conditions (Fig. 3). This applied to M.EcoVIII and M.HindIII, but not to M.LlaCI. Similar results were obtained for a hemimethylated substrate which under star activity conditions was modified extensively by M.HindIII, but not by M.EcoVIII and M.LlaCI (Fig. 3). This prompted us to test whether the increased methylation level under star activity conditions is due to the off-target modification at the flanking regions of the target site. Thus, we used two ds oligonucleotides (Control L1/L2 and Control R1/R2; Table 1), which were deprived of the target sequence (AAGCTT), but contained a nucleotide sequence specific either for the left or for the right arm of oligo AAGCTT1/AAGCTT2. As a result, we have found that none of these oligos were modified at a level higher than 1% (in comparison to oligo AAGCTT1/AAGCTT2), neither under standard nor star conditions (data not shown). Thus, we concluded that under standard conditions the target site localized at the synthetic DNA molecule may not be fully methylated. As it was shown previously, synthetic DNA varies from a natural molecule. Differences may concern either electrostatic properties of the substrate, which are crucial in DNA-protein interactions^28^, or the substrate structure, which is less ordered in case of synthetic oligonucleotides. Both factors may affect formation of highly ordered DNA-protein complexes^29^.

We have also tested the ability of isospecific MTases to modify single-stranded DNA. We found that M.EcoVIII, contrary to M.HindIII and M.LlaCI, was able to modify the target site localized within ssDNA (Fig. 3A). The level of ssDNA methylation was 7-fold lower than dsDNA. However, upon star activity conditions (30% DMSO), the efficiency of ssDNA modification was raised by up to 50% of the relative activity, as shown in Fig. 3A.

### Isospecific MTases modify the off-target sites, but not to the same extent

**Figure 4:**
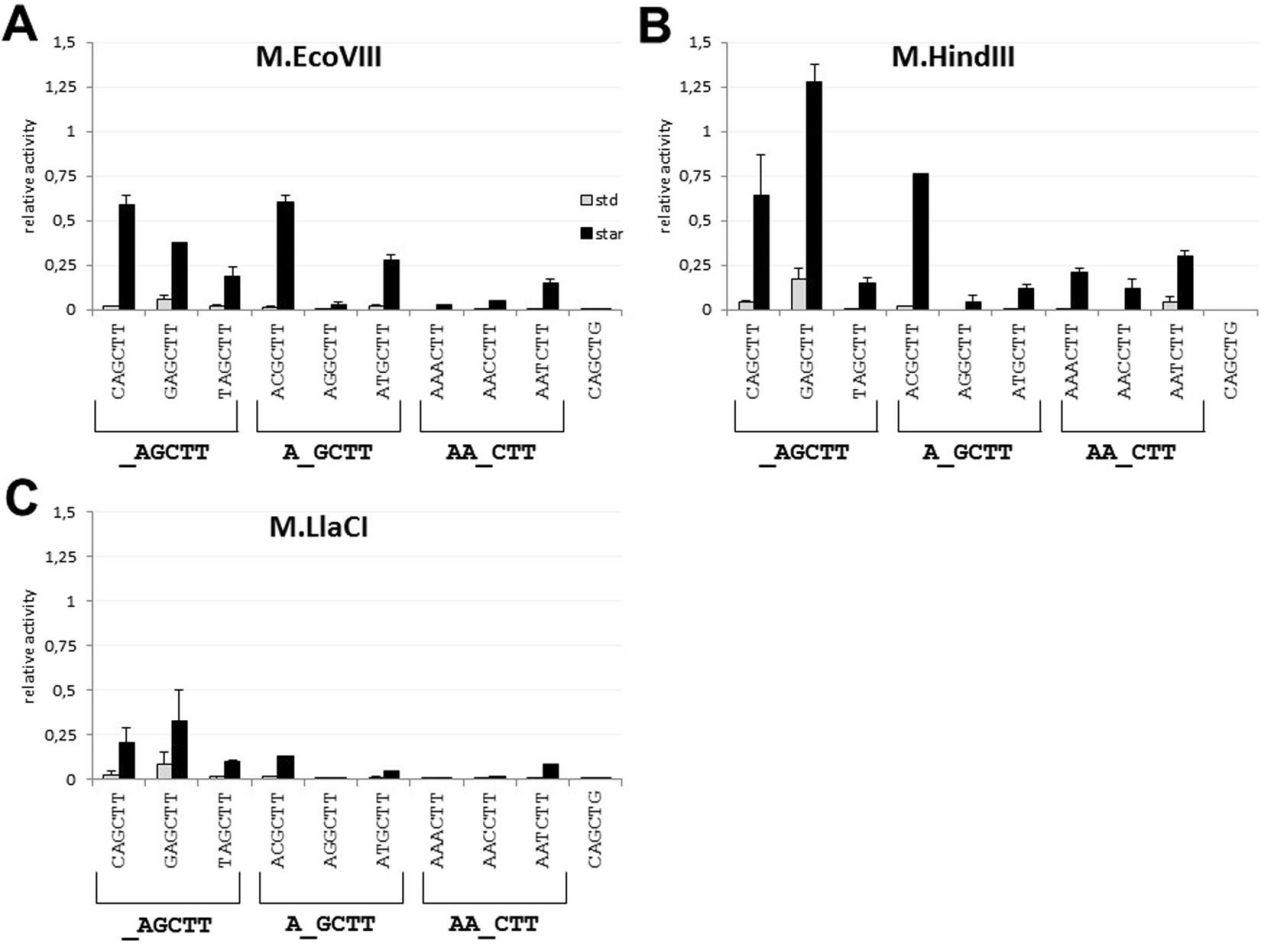
Analysis of DNA methyltransferase activity towards an oligo with secondary sites – sequences with substitution with respect to the 5′-AAGCTT-3′ specific sequence in position 1^th^–3^rd^. Methylation was carried out by M.EcoVIII (**A**), M.HindIII (**B**), M.LlaCI (**C**) in standard (grey columns) or star activity (black columns) reaction mixtures. Methylation level was normalized to the one obtained in standard reaction mixture with the 5′-AAGCTT-3′ canonical sequence (methylation level = 1).

Many MTases under star activity conditions modify secondary target sites showing pronounced similarity to the specific sequence. In most cases, off-target sites differ by one nucleotide from the specific sequence^6^. Thus, in the next step we tested whether the isospecific MTases may differ in modification of such secondary sequences. We used 29-mer oligonucleotides with all possible single substitutions in the first half of the 5′-AAGCTT-3′ target sequence (position 1^st^–3^rd^; Table 1). The flanking sequences surrounding off-target sites remained unchanged. Each time the modification level was normalized to the level obtained for unmethylated target sequence (ds oligo AAGCTT1/AAGCTT2). We found that under standard conditions, the only modified off-target sequence was 5′-GAGCTT-3′ (substitution underlined), methylated by all three MTases at the level of 6–17% (Fig. 4). On the other hand, under star activity conditions we observed extensive modification of the off-target sequences, especially for M.EcoVIII and M.HindIII (Fig. 4A,B). M.LlaCI modified such sequences to the much lesser extent (Fig. 4C). Out of nine sequences tested, three off-target sequences were methylated preferentially by all isospecific MTases: 5′-CAGCTT-3′, 5′-GAGCTT-3′ and 5′-ACGCTT-3′. M.EcoVIII modified 5′-CAGCTT-3′ and 5′-ACGCTT-3′ at the same level, while methylation of 5′-GAGCTT-3′ was slightly reduced. In case of M.HindIII and M.LlaCI, we observed preferences towards 5′-GAGCTT-3′ over 5′-ACGCTT-3′ and 5′-CAGCTT-3′ (Fig. 4B,C). In general, off-target sequences with substitutions at the third position were the worst substrates for all enzymes except M.HindIII, where all three possible secondary sites were at least partially modified (Fig. 4B). An opposite effect was observed for M.LlaCI which had the lowest tolerance to off-target sequences. Among all sites tested, only the 5′-GAGCTT-3′ sequence was modified at a level of 35% (Fig. 4C).

### Isospecific MTases show similar tolerance to the mismatches within the target site

**Figure 5:**
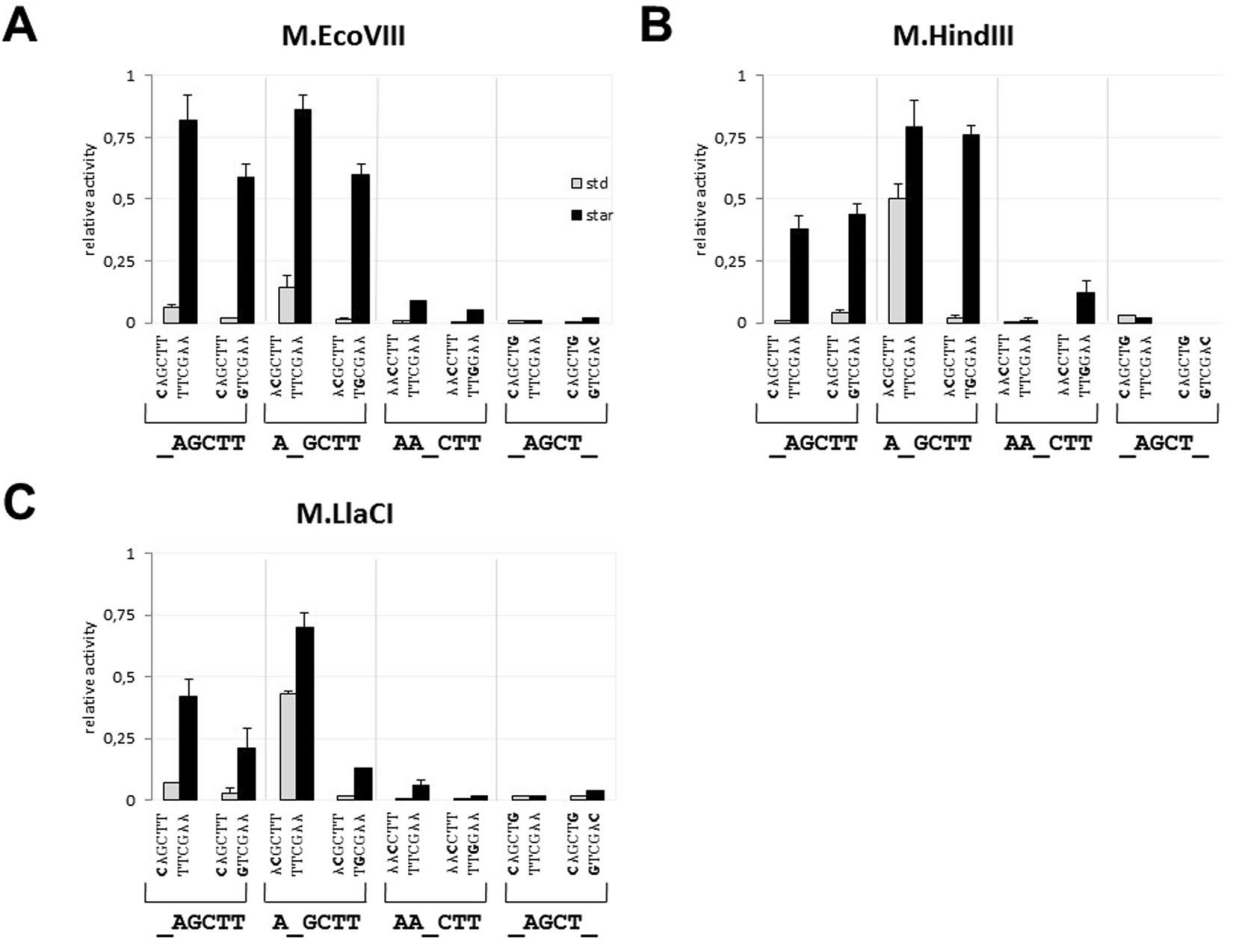
Enzymes′ specificity towards mismatch or secondary sequences. Substitutions concerned the 1^st^–3^rd^ position with respect to the 5′-AAGCTT-3′ specific sequence. Methylation was carried out by M.EcoVIII (**A**), M.HindIII (**B**), M.LlaCI (**C**) in standard (grey columns) or star (black columns) reaction mixtures. Methylation level was normalized to the one obtained in standard reaction mixture with the 5′-AAGCTT-3′ canonical sequence (methylation level = 1). Substrates were constructed by hybridization of oligo AAGCTT2 with CAGCTT1, ACGCTT1, AACCTT1 or CAGCTG1 (Table 1).

In the next step, we tested activity of isospecific MTases towards ds oligonucleotides with one or two imperfect or perfect mismatches in the target site. We decided to substitute nucleotides at selected positions (1^st^–3^rd^) with cytidine, guided by reports showing ability of some adenine-*N*^6^ MTases to modify a cytosine residue localized at the target site to *N*^4^-methylcytosine, especially in a mismatched context^5,30^. In addition, the cytosine-*N*^4^ MTase PvuII was found to exhibit a promiscuous activity also modifying adenine residue in the target site^7,31^. Thus, the following off-target sequences were tested: 5′-CAGCTT-3′, 5′-ACGCTT-3′, 5′-AACCTT-3′ and 5′-CAGCTG-3′ (substitution is underlined). Accordingly, the complementary sequence for imperfect mismatches was 5′-AAGCTT-3′, while complementary sequences for perfect mismatches were: 5′-AAGCTG-3′, 5′-AAGCGT-3′, 5′-AAGGTT-3′ and 5′-CAGCTG-3′. In general, the tested enzymes preferred substrates carrying target site with imperfect mismatches. Methylation activity was much more efficient under star activity than standard conditions (Fig. 5). However, the oligo with substitution at the 2^nd^ position (5′-ACGCTT-3′) was modified in up to 50% by all three MTases. On the other hand, under star activity conditions the ds oligo carrying the same sequence was methylated in almost 80%. In addition, the enzymes preferred the sequences with the mismatch at the 1^st^ and 2^nd^ positions over the 3^rd^ position (Fig. 5). None of the tested MTases was able to methylate nucleotide sequence containing two mismatches (5′-CAGCTG-3′), even under star activity conditions. Therefore, we concluded that the analyzed MTases do not modify cytosine residue either under standard or star activity conditions. However, we cannot exclude possibility that observed effect is due to weak recognition of such sequences, than to the enzymes′ inability to methylate a cytosine residue. Moreover, under standard conditions the target site with substitution at the 1^st^ position (5′-CAGCTT-3′) was a worse substrate than the sequence with substitution at the 2^nd^ position (5′-ACGCTT-3′). Similarly, under star activity conditions the 5′-ACGCTT-3′ sequence was modified at the highest level by all tested MTases, however, M.EcoVIII also efficiently methylated the 5′-CAGCTT-3′ site (Fig. 5).

### Analyzed MTases differ in unspecific interactions with the DNA phosphate backbone

**Figure 6:**
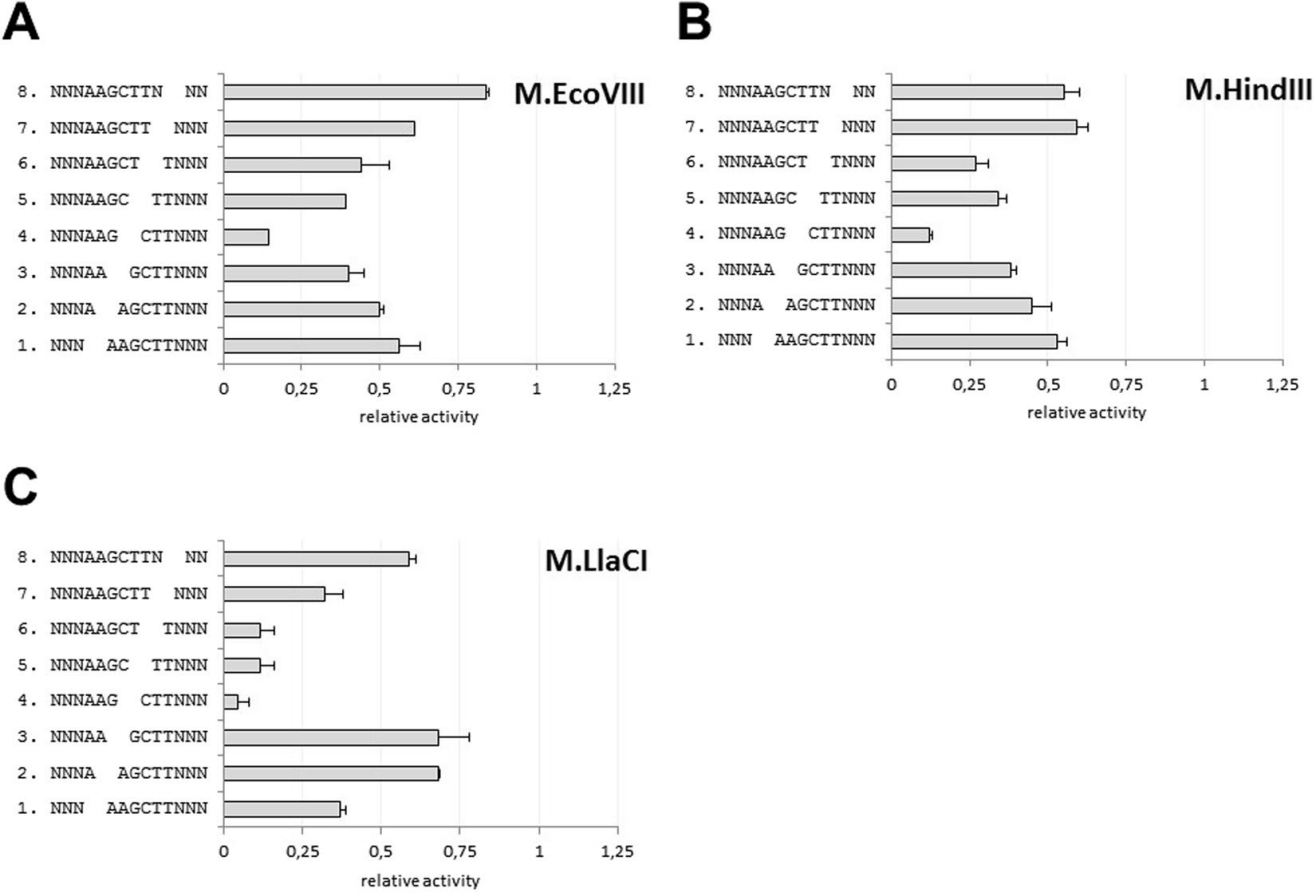
Modification of the 5′-AAGCTT-3′ specific sequence devoid of a single phosphodiester bond within the sequence or in a close proximity to it. Methylation was carried out by M.EcoVIII (**A**), M.HindIII (**B**), M.LlaCI (**C**) in standard reaction mixtures. Methylation level was normalized to the one obtained in a standard reaction mixture with the 5′-AAGCTT-3′ target sequence (methylation level = 1). In each case, the oligonucleotides are double stranded, though only the upper strand is shown for clarity. Gap represents the lack of a phosphodiester bond. Substrates were made by hybridization using three oligos as described in Methods and shown in Fig. 2.

Next, we addressed the importance of unspecific interactions within the phosphate backbone of the target sequence. We used the series of substrate ds oligos devoid of a single phosphodiester bond within one strand of the target sequence or in a very close proximity to it. Interactions with phosphodiester bonds within the target sequence are of a remarkable importance, as reported in the case of M.BamHI^32^. We predicted the gap effect to be significant if the methylation level is less than 0.5, meaning the modification of both target adenine residues is affected. We considered the level of modification to be more than 0.5 when the two target adenine residues were at least partially modified. Our data revealed that for each tested enzyme the most crucial is the central phosphodiester bond (G^3^-C^4^) of the target site (Fig. 6). Its absence resulted in 7–22 fold decrease in modification as compared to the control dsDNA substrate. Moreover, we have found that absence of a single phosphodiester bond within the target sequence reduces methylation to 0.04–0.68, with the most severe effect for M.LlaCI (Fig. 6). For M.EcoVIII, the gap effect seems to be symmetrical, i.e. the closer to the target site center, the more reduced methylation. The absence of phosphodiester bond between proximal nucleotides of the target sequence and flanking nucleotides (N-A^1^ and T^6^-N, respectively) has a comparable effect on methylation, with the level decreased to 60%, and for A^1^-A^2^ or T^5^-T^6^ bonds the level reduced to about 50% (Fig. 6). We also have similar observations for M.HindIII, however, in this case the symmetry is not as clear as for M.EcoVIII. On the other hand, M.LlaCI seems to act in a different manner. It seems the essential contacts are G^3^-C^4^, as well as C^4^-T^5^ and T^5^-T^6^, which all reveal substantial negative effect on methylation (9–22-fold). The results obtained led us to conclude that M.LlaCI may not interact with the target adenine 3′ phosphate, as substrate lacking A^1^-A^2^ phosphodiester bond is modified at 68%, which suggests that both target adenines are at least partially methylated. The same A^1^-A^2^ contacts seem to be critical for M.EcoVIII and M.HindIII, as its lack affects modification of both target adenine residues (Fig. 6).

### Isospecific MTases prefer substrates with target site localized at some distance from the end of DNA fragment

**Figure 7:**
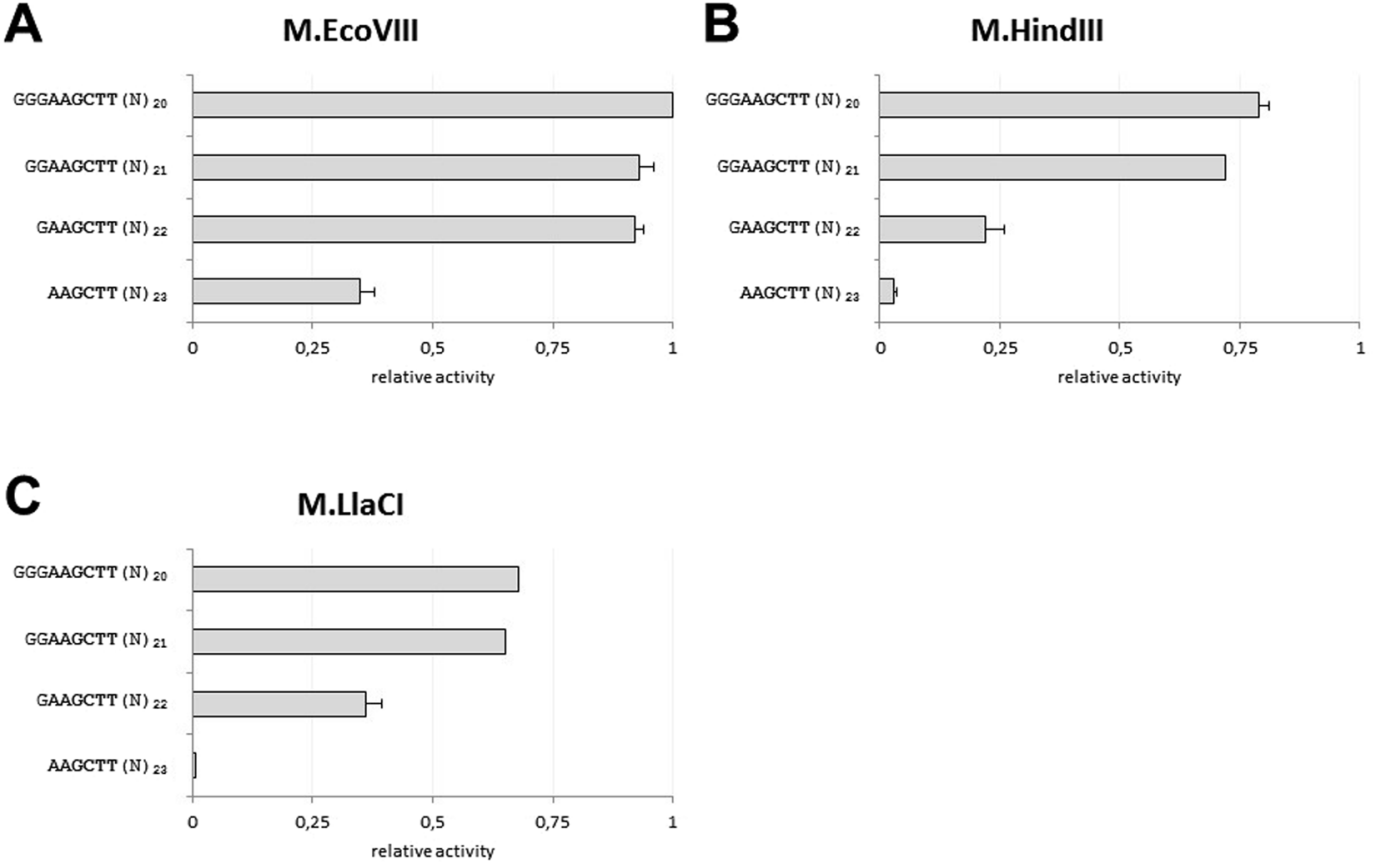
Modification of the specific sequence 5′-AAGCTT-3′ localized at various distances from the oligo′s end. Methylation was carried out by M.EcoVIII (**A**), M.HindIII (**B**), M.LlaCI (**C**) in standard reaction mixtures. Methylation level was normalized to the one obtained in a standard reaction mixture with the 5′-AAGCTT-3′ canonical sequence localized 10 nt from the 5′ end and 13 nt from the 3′ end (methylation level = 1).

In the next step, we compared activity of all three isospecific MTases using as substrates ds oligos with target site localized in close proximity to the end of the substrate DNA (Fig. 7). We found that all of the analyzed MTases are sensitive to the position of the target site. The most dramatic effect was observed when the target site was at the very end of the DNA molecule (Fig. 7B,C). With one base pair that separates the target site from the end of DNA substrate we observed a higher enzymatic activity. This is the most evident in case of M.EcoVIII (Fig. 7A). However, in case of M.HindIII and M.LlaCI, modification of the DNA substrate with target site localized 3 base pairs from the oligo end resulted in 79% and 68% relative activity, respectively (Fig. 7B,C). This led us to conclude that despite being isospecific, all three MTases interact with the substrate DNA in a different way.

### Determination of the kinetic parameters for M.EcoVIII

Steady-state kinetic parameters of M.EcoVIII were determined using ds oligos as substrates (Table 3). The initial rates of methylation were first order, and Michaelis-Menten kinetics were obeyed with respect to both, AdoMet and DNA. The *K*_m_ for the methyl group donor (AdoMet) was 0.27 ± 0.006 µM, and for determination of kinetic parameters for DNA substrates we used AdoMet at a 6.5-fold excess (1.76 µM). M.EcoVIII preferred the hemimethylated specific sequence over unmethylated one, with the relative k_cat_/K_m_ about 4.4-fold. In addition, M.EcoVIII appeared to methylate the specific sequence localized at ssDNA (Fig. 3). Kinetic measurements yielded values of K_m_ = 257.6 µM and k_cat_ = 0.03, and efficiency of methylation was ten times lower than in the case of dsDNA, which is in agreement with results obtained previously^27^. The efficiency of the secondary site methylation was also lower by one order of magnitude, and ranged from 0.17 to 0.43 × 10^4^ s^−1^ M^−1^ (Table 3). The DNA substrate preference for M.EcoVIII may be ordered as follows:

**Table 3:** Steady state kinetics parameters for M.EcoVIII.

ds oligos	Specific sequence	substitution	K_m_ [nM]	k_cat_ [min^−1^]	k_cat_/K_m_ × 10^4^ [s^−1^ M^−1^]	Relative k_cat_/K_m_
AAGCTT1 + AAGZTT2	AAGCTT TTCGAA	target site	75.1 ± 6.5	0.088 ± 0.002	1.96 ± 0.08	1
MAGCTT + AAGCTT2	MAGCTT TTCGAA		24.4 ± 3	0.126 ± 0.002	8.61 ± 0.09	4.39 ± 0.086
AAGCTT1	AAGCTT		257.6 ± 0.01	0.030 ± 0.002	0.19 ± 0.01	0.1 ± 0.002
CAGCTT1 + GAGCTT2	CAGCTT GTCGAA	A^1^ → C	78.2 ± 3	0.020 ± 0.001	0.42 ± 0.04	0.21 ± 0.014
GAGCTT1 + GAGCTT2	GAGCTT CTCGAA	A^1^ → G	78.4 ± 9	0.020 ± 0.002	0.43 ± 0.08	0.22 ± 0.035
TAGCTT1 + TAGCTT2	TAGCTT ATGCAA	A^1^ → T	85.5 ± 3	0.013 ± 0.002	0.22 ± 0.05	0.11 ± 0.023
ACGCTT1 + ACGCTT2	ACGCTT TGCGAA	A^2^ → C	89.0 ± 4	0.013 ± 0.002	0.26 ± 0.03	0.13 ± 0.012
AGGCTT1 + AGGCTT2	AGGCTT TCCGAA	A^2^ → G	91.2 ± 3	0.012 ± 0.003	0.17 ± 0.01	0.09 ± 0.003
ATGCTT1 + ATGCTT2	ATGCTT TACGAA	A^2^ → T	91.4 ± 2	0.015 ± 0.001	0.27 ± 0.03	0.14 ± 0.011
AAACTT + AAACTT2	AAACTT TTTGAA	G^3^ → A	102.3 ± 3	0.020 ± 0.003	0.17 ± 0.07	0.09 ± 0.034
AACCTT1 + AACCTT2	AACCTT TTGGAA	G^3^ → C	127.3 ± 0	0.016 ± 0.002	0.19 ± 0.01	0.10 ± 0.007
AATCTT1 + AATCTT2	AATCTT TTAGAA	G^3^ → T	143.0 ± 7	0.020 ± 0.004	0.29 ± 0.06	0.15 ± 0.027

M- methylated adenine.

^hemi^AAGCTT > AAGCTT > GAGCTT ≥ CAGCTT > AATCTT > ATGCTT > ACGCTT > TAGCTT > AACCTT = ssAAGCTT > AGGCTT = AAACTT. The revealed substrate preference was in accord with data presented at Figs 3 and 4. Surprisingly, the 5′-GAGCTT-3′ secondary target site was the only sequence at which modification was detectable under standard reaction conditions. The kinetics analysis showed that indeed this substrate was modified with the highest efficiency among all tested secondary sequences. Moreover, we obtained a relatively high modification for 5′-CAGCTT-3′, as well as for 5′-AATCTT-3′, 5′-ATGCTT-3′ and 5′-ACGCTT-3′. In agreement with previous results, the following sequences were methylated with the lowest efficiency: 5′-AAACTT-3′ and 5′-AGGCTT-3′.

## Discussion

The aim of our study was to characterize three isospecific type II MTases in order to define their substrate preferences. We found that the analyzed enzymes preferred a hemi-methylated oligonucleotide over unmethylated one. M.EcoVIII steady-state kinetics showed that such DNA is modified over 4-fold more efficiently in comparison with unmethylated ds oligo used as a substrate. This is in agreement with the function of DNA MTases that are part of RM systems, as they provide genomic DNA with a distinctive label that enables to distinguish between self- and non-self DNA^1,33^. Moreover, M.EcoVIII, but not M.HindIII and M.LlaCI, exhibits ability to methylate ssDNA, although 10-fold less efficiently than dsDNA^27^. Specific modification of ssDNA has been reported for a few MTases so far, e.g. the P1 prophage-encoded type III M.EcoP1I. This MTase modifies ss- and dsDNA (similar K_m_ and k_cat_) with comparable efficiency and it was shown that ssDNA methylation is not linked with formation of double stranded structures^34,35^. Such unusual phage MTase feature may be related to the survival of single-stranded DNA phages in bacterial host^35^. In addition, M.DpnA and M.BcnIA are capable to modify ssDNA with efficiency greater or similar to that of dsDNA. They belong to type II RM systems consisting of restriction endonuclease and two DNA MTases with the same substrate specificity^36,37^. The biological significance of such enzymes may be associated with protection against plasmid transmission during transformation, as it proceeds by an ssDNA intermediate^36,38^. In case of M.EcoDam and M.BamHI, which were also found to be able to modify ssDNA, it was suggested that an enzyme’s interaction with its substrate is associated with formation of double-stranded structures containing the target site flanked by ssDNA regions^32^. Similar observation was made in the case of eukaryotic DNMT1^39^, where ssDNA substrate without the target palindrome was not modified^40^. M.EcoVIII could possibly pair ssDNA substrate, although two other MTases, LlaCI and HindIII, do not exhibit such propensity towards ssDNA.

DNA MTases are regarded as highly specific enzymes. However, the off-target modifications were also reported, presenting a whole range of activities *in vitro*, from a high fidelity (like M.AluI) to the most prone to methylate the off target sites like, M.HhaI or M.HaeIII^6,8,41^. Though the translation of data from *in vitro* to the *in vivo* conditions is always a challenge, recent technologies confirmed the star-activity was also detected in the living cells. The SMRT sequencing method and methylated base analysis allowed to detect a significant number of off-target modification *in vivo* for M.EcoKDam^11^ and M.HaeIII^6^. In the course of evolution of the RM systems, changing the specificity of an ENase needs to be preceded by a similar process concerning cognate DNA MTase. This is extremely important considering toxic effect exerted by a restriction enzyme on the host cell. Ability to modify secondary sequences seems to be an indispensable step in evolution of the RM systems toward enzymatic units with novel specificities^6,42^.

In order to determine the spectrum of M.EcoVIII off-target substrate specificities, a detailed analysis of the enzyme kinetics was performed using ds oligos with secondary target sites that differed by one-base-pair from the specific sequence as substrates. Efficiency of methylation of such substrates was about 4–11 fold lower than the one for the target site. In case of M.EcoRI, similar analysis revealed a 5–23000 fold reduction in methylation efficiency when off-target sites with substitutions at position 1^st^–2^nd^ or 5^th^–6^th^ with respect to the specific 5′-GAATTC-3′ sequence were used^43,44^. Another exemplary enzyme is M.FokI which possesses two domains, each one recognizing and modifying the adenine residues within complementary strands of the 5′-GGATG-3′/3′-CCTAC-5′ asymmetric specific sequence^45,46^. It was shown that M.FokI N-terminal domain (target sequence 5′-GGATG-3′) methylated only one (5′-AGATG-3′) out of twelve tested secondary sites, and the efficiency was greatly reduced (69-fold). Interestingly, the C-terminal domain (target sequence 5′-CATCC-3′) was able to methylate ten out of twelve secondary sites, and surprisingly modification of 5′-TATCC-3′ and 5′-CATCT-3′ sites was 3 and 5.6-fold reduced in comparison to the target sequence, respectively. Moreover, the C-terminal domain also modified a double substitution in the specific sequence 5′-GATGC-3′^41^. Our kinetics data indicate, that M.EcoVIII exhibits a relatively relaxed specificity under standard conditions, with the highest preference towards the 5′-GAGCTT-3′ and 5′-CAGCTT-3′ sites (4.5 and 4.8-fold methylation decrease in comparison to the specific sequence, respectively). Off-target sites with substitutions at the 2^nd^ position were methylated with similar relative efficiency that ranged between 0.09–0.14. Among them, the sequence where A:T pair was changed to G:C was one of the three sites modified to the lowest extent (the other sites were 5′-AACCTT-3′ and 5′-AAACTT-3′). M.EcoVIII shows low tolerance to targets with substitutions at the 3^rd^ position. Secondary sites, such as 5′-AANCTT-3′ were modified with the relative efficiency decreased by an order of magnitude. The only exception is the 5′-AATCTT-3′ sequence, where we observed 6.7× decrease in methylation. The guanine residue may be recognized by an enzyme by contacts due to N^7^ and O^6^ in the major groove and the amino group in the minor groove. O^4^ of thymine locates in an analogous position to O^6^, but G → T conversion removes any possible contacts with N^7^, moreover, the methyl group and O^2^ may create a steric barrier. G → C also removes contacts with N^7^, and what is more, cytosine does not offer any acceptor of hydrogen bond in the major groove, while in the minor groove instead of a donor it has an acceptor (O^2^). G → A substitution preserves contacts with N^7^, but instead of the acceptor (O^6^) adenine possesses a donor at N^6^ and removes the amino group from the minor groove. Analysis of interactions between DNA MTase and target sequence shows that enzymes do not contact each functional group of a base. For instance, M.EcoRI does not interact with the methyl group of the second thymine (5′-GAATTC-3′)^47^, and contacts with cytosine and guanine are formed through the minor groove^48,49^. Thus, the specific sequence recognition mechanism is very complex and cannot be predicted by simple analysis of the available donors/acceptors. Relatively inefficient methylation of 5′-AANCTT-3′ may result from the specific site symmetry disturbance caused by introducing a new base in a proximity of the site′s axis. Since in the analyzed sequences there are two target adenines, introducing substitution at the 1^st^ position (5′-NAGCTT-3′) did not preclude interaction with the second adenine residue in the opposite strand. The closer distance of the changed base to the center of the sequence, the interaction with such degenerate site is more hindered. Seemingly different result was obtained for T4Dam (5′-GATC-3′), where lack of the target adenine residue in one strand prevented methylation of such substrate even if the second target residue was untouched^50^. However, since the target residue is placed in the middle of the sequence, thus its absence would lead to significant changes in the symmetry of the specific sequence. Similar observations concerned M.BamHI and M.EcoDam – any disorders in the specific sequence, especially near the center, caused a decrease in the methylation efficiency^32^. In case of M.EcoVIII, the differences in methylation efficiency did not result from changes in affinity (K_m_) but rather in reduction in k_cat_, which may suggest that the readout mechanism arises from proper positioning of the catalytic residues rather than binding, analogically to what was proposed for M.EcoRI^43^.

**Figure 8:**
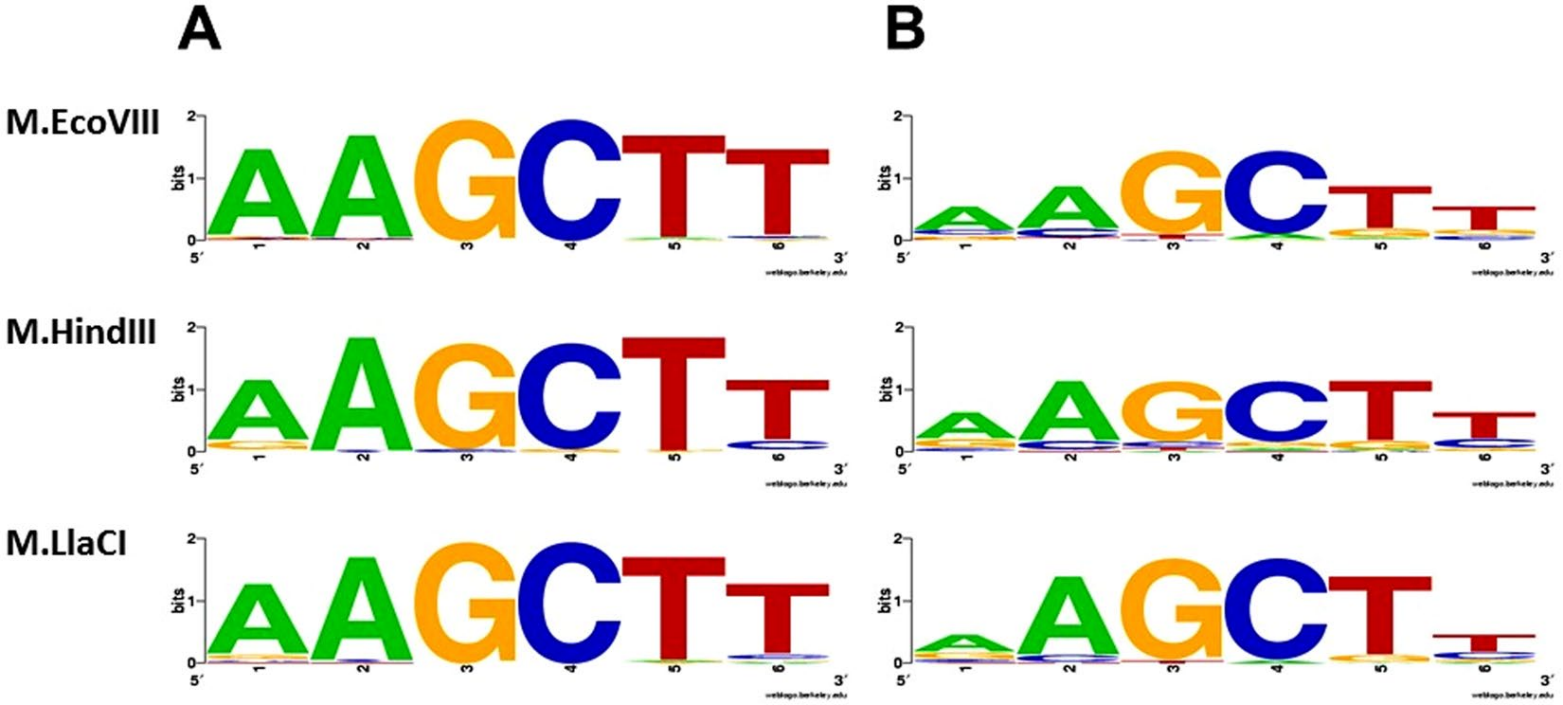
LOGOs of sequences modified by the studied enzymes under standard (**A**) or star activity conditions (**B**).

**Figure 9:**
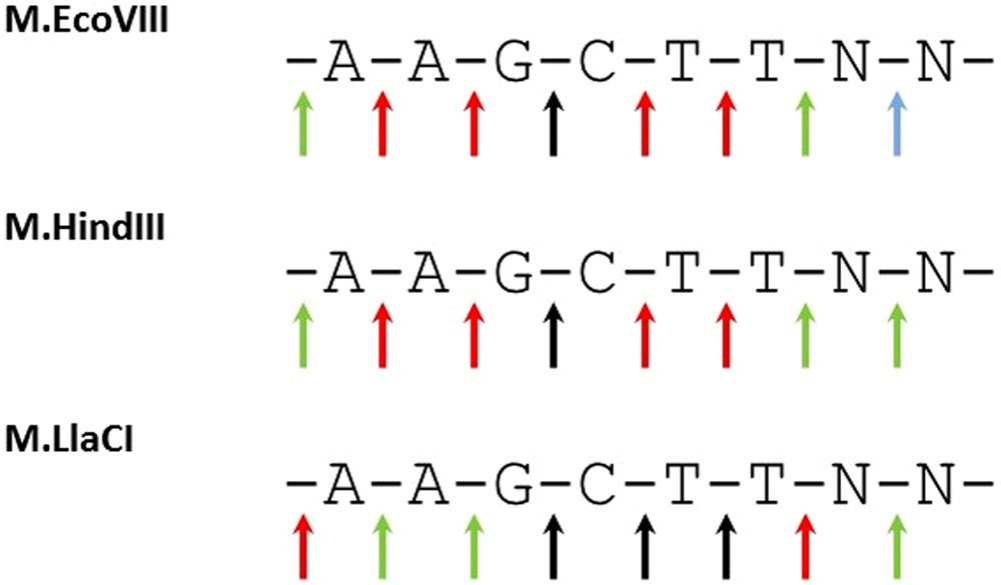
Impact of interaction with a particular phosphate on modification carried out by AAGCTT specific DNA methyltransferases. Black arrows indicate the most important interaction (over 75%), red arrows – 75–50%, green arrows – 50–25% and blue arrow – below 25%.

All DNA MTases tested here were able to methylate off-target sites when reaction was supplemented with specificity modifying agents. Results obtained for M.EcoVIII kinetics revealed that hierarchy of substitutions at each position is similar to that obtained with DMSO. Therefore, one can assume that agents leading to star activity emphasize tendency of an enzyme to recognize a particular sequence rather than change it. M.EcoVIII and its isomethylomers exhibit lower specificity towards external nucleotides of the canonical sequence. This can be seen particularly in the LOGO (Fig. 8) generated on the basis of results presented on Fig. 4. Since under standard reaction conditions virtually no sequence was modified at a detectable level, the LOGOs look similar to one another (Fig. 8A). Bigger differences were obtained under star activity conditions (Fig. 8B). The studied enzymes are by far more flexible towards substitutions in the extreme positions, while nucleotides in the central part form a core where the interactions are essential. What is more, M.LlaCI shows only a low tendency to methylate off-target sites. This may result from a possible dimerization^26^. In experiments with secondary sites, we used unmethylated substrates so the enzyme had a chance to interact with a bare target site. However, hypothesis about M.LlaCI acting as a dimer needs further experimental verification. Crystallographic analysis of MTases in complex with substrate DNA indicate that interaction with the target sequence concerns either the bases or the sugar-phosphate backbone, even outside the specific sequence^51^. However, interactions with particular phosphate bonds within the specific sequence are not equally important. Despite recognizing the same 5′-AAGCTT-3′ nucleotide sequence, the group of enzymes tested here achieve it in a different way (Fig. 9). It seems that for M.LlaCI, interaction with the target adenine 3′ phosphate is not crucial, yet this enzyme is by far more sensitive to the lack of phosphate outside the specific sequence (Fig. 9, oligo 8). This interaction is also important for M.HindIII, but not for M.EcoVIII. On the other hand, M.EcoVIII and M.HindIII interact with both target adenine phosphates. Comparison of M.EcoVIII and its isomethylomer data with the one obtained for T4Dam or M.BamHI, reveals a surprising difference in effect caused by lack of the phosphodiester bond between nucleotides vis-à-vis the target base. We observed a severe decrease in modification of the substrate oligo without the phosphodiester bond between two thymine residues (-A-A-G-C-T T-). For all tested enzymes it hindered methylation of the two target adenines. However, it was reported that lack of the T-C phosphate in case of T4Dam (-G-A-T C-, with adenine residue being methylated) led only to 1.5× drop in k_cat_, while K_m_ did not change. Similarly, oligo without the G-A bond (-G-G A-T-C-C-, with the first cytosine residue being methylated) caused only 12% reduction in methylation carried out by M.BamHI; when substrate without the G-G bond was used, the methylation level was even higher in comparison to control with all bonds^32^. Thus, it seems that T4Dam and M.BamHI interact with the specific sequence phosphate backbone differently than M.EcoVIII and its homologs.
